# Primary Leptomeningeal B-cell Lymphoma in an Immunocompetent Adult: Case Report

**DOI:** 10.7759/cureus.19619

**Published:** 2021-11-16

**Authors:** Andrea Calderon-Castro, Leonardo Enciso, Rafael Tejada-Cabrera

**Affiliations:** 1 Neurology, Hospital Universitario Nacional de Colombia, Bogotá, COL; 2 Hematology, Hospital Universitario Clínica San Rafael, Bogotá, COL; 3 Hematology, Hospital Universitario de La Samaritana, Bogotá, COL; 4 Hematology and Oncology, Hospital Universitario Nacional de Colombia, Bogotá, COL

**Keywords:** methotrexate, flow cytometry, meningeal neoplasms, b-cell lymphoma, lymphoma

## Abstract

Primary leptomeningeal lymphoma (PLML) is a rare disease, comprising less than 1% of all lymphomas. Clinical manifestations include headache, encephalopathy, ataxia, cranial nerve palsy, and myelitis. Diagnosis requires a combination of magnetic resonance images (MRI), cytology, flow cytometry of cerebrospinal fluid (CSF), and an extensive workup to rule out systemic lymphoma.

We describe the case of a 49-year-old man who developed subacute onset headache, encephalopathy, and blindness. Whole-body examinations, including a bone marrow trephine biopsy, excluded systemic lymphoma. Brain MRI showed leptomeningeal enhancement. Cytology and flow cytometry of CSF found a clonal B-cell population making a diagnosis of PLML. He began treatment with rituximab and high-dose methotrexate (HD-MTX), with progressive clinical improvement. CSF analysis after two cycles and one intrathecal methotrexate dose was normal.

Brain and spinal MRI images plus CSF analysis, along with an extensive workup to exclude systemic lymphoma, are necessary to diagnose PLM. Early treatment with HD-MTX alone or in combination with rituximab improves clinical outcomes.

## Introduction

Lachance et al. coined the term primary leptomeningeal lymphoma (PML) in 1991 to describe a malignant disease characterized by meningeal infiltration of tumoral lymphoid cells, without cerebral parenchyma or systemic evidence of lymphoma [1]. It is a rare disease representing less than 1% of all lymphomas and 7% of primary central nervous system lymphomas (PCNSL) [2]. In a large reported series [2], the median age at diagnosis was 51 years, and it was more frequent in men. Clinical manifestations include headache, seizures, blindness, diplopia, encephalopathy, ataxia, cranial nerve palsy, and other neurological symptoms [2].

The diagnosis requires a high index of suspicion and a combination of images, cytology, flow cytometry, and, in some cases, meningeal biopsy [3]. Neuroimaging, including MRI, could be normal or may show leptomeningeal enhancement or hydrocephaly [2]. Cerebrospinal fluid (CSF) cytology and flow cytometry will detect a clone of malignant lymphocytes [3]. Most cases show a mature B-cell phenotype, with mature T-cell and immature B-cell being less frequent [2]. Treatment options include chemotherapy with high-dose methotrexate (HD-MTX) alone or in combination with other agents and radiotherapy in selected cases.

Here we report a case of a 49-year-old man with PLM treated with HD-MTX in combination with rituximab, with a rapid response and clearance of tumor cells.

## Case presentation

A 49-year man presented to the emergency department with subacute onset headache, nausea, and diplopia. Medical history included a depressive disorder and occasional alcohol consumption.

He reported episodic night sweats and weight loss of less than 10% of his usual weight. He did not report any other symptoms, including cervical, axillary, and inguinal lymphadenopathy. The neurological examination in the emergency department found a disoriented patient with isochoric pupils and no focal neurologic deficit.

The first brain MRI was considered normal, but the initial cerebrospinal fluid (CSF) sample was abnormal, with lymphocytic pleocytosis, high protein, and hypoglycorrhachia (Table 1, Column A).

**Table 1:** Cerebrospinal fluid analyses ADA: Adenosine Deaminase; PCR: Polymerase Chain Reaction

CSF Sample	A	B	C	D	E
	October 9^th^	October 11^th^	October 27^th^	December 6^th^	December 27^th^
Leukocytes (cells/ul)	20	189	124	300	335
Lymphocytes (%)	59	44	10	3	100
Polymorphonuclear (%)	41	56	90	1	0
Red blood cells (cells/ul)	0	20	4	0	0
Glucose (mg/dL)	27	21,63	30	28,36	37,09
Protein (mg/dL)	113	124	89,3	68,71	48
Gram coloring	Negative	Negative	-	Negative	Negative
Culture for common germs	Negative	Negative	-	Negative	Negative
Film array	Negative	Negative	-	-	-
Chinese Ink	Negative	Negative	-	Negative	Negative
Cryptococcus antigen	Negative	Negative	-	Negative	Negative
Ziehl Neelsen	Negative	Negative	-	Negative	Negative
Mycobacterium tuberculosis PCR		Negative	Negative	Negative	-
Epstein Barr virus PCR	-		-	Negative	-
ADA	-	8,89	10,3	17,34	-

Flow cytometry found pathological large CD19/CD20 and lambda monoclonal B lymphocytes suggestive of lymphoma (Table 2, Column A). An extensive workup, including CT scans and bone marrow biopsy, was negative for systemic lymphoma.

**Table 2:** Cerebrospinal fluid flow cytometry

CSF Sample	A	B	C	D
	October 27^th^	December 6^th^	December 17^th^	January 5^th^
Total cells (cells/uL)	262.2	52.9		11.97
Neutrophils (%)	3.55	0.8		
Monocytes (%)	6.3	3.8		
T Lymphocytes (%)	32.25	5.4	7,27	97.89
CD4+ (%)	25.31	2.9	3.45	50.7
CD4+ CD8+ (%)	0.16	0.1	0.07	1.14
CD8+ (%)	5.89	2.2	3.35	
CD4- CD8- (%)	0.9	0.5	0.13	2.82
Mature B lymphocytes (%)	3.2			0
Kappa + (%)	1.6			
Lambda + (%)	1.6			
Pathological B lymphocytes	(n=143.4) 54.7%	(n=47.2) 89.3%	92.49%	Not observed
Size	Large	Large	Large	
CD 19	Positive	Positive	Positive	
CD10	Negative	Negative		
CD45	Positive	Positive	Positive	
CD20	Weakly positive	Positive	Positive	
CD38	Positive	Positive	Positive	
Kappa	Negative	Negative	Negative	
Lambda	Positive	Positive	Positive	
CD4	Negative	Negative	Negative	
CD8	Negative	Negative	Negative	
CD56	Negative	Negative	Negative	
CD5	Negative	Negative	Negative	
CD200	Negative	Negative	Negative	
CD14	Negative	Negative	Negative	

A new CSF analysis found persistent pleocytosis and high adenosine deaminase (ADA) activity (Table 1, Columns B-C). The patient started treatment with isoniazid, rifampin, and ethambutol and received it for 50 days without clinical improvement. Cultures and protein chain reaction (PCR) of CSF for mycobacterial and other infections were negative.

Because of clinical deterioration due to altered consciousness and quickly progressive blindness is admitted to our center. 

At admission, the patient had stable vital signs with a Karnofsky performance status of 70. Clinical examination found a disoriented patient with an acute ill appearance. A neurologic exam revealed a stiff neck and no light perception in either eye, with preserved direct and indirect light reflexes. Pupils were symmetrical, and fundoscopy was normal. Strength was 5/ 5 in four extremities (Medical Research Council Scale), and deep reflexes were normal. Nodal areas were usual with no lymphadenopathy or splenomegaly. Skin and testis do not have abnormalities.

Whole-body enhanced computed tomography and testicular ultra-sound were negative for nodal or extra-nodal involvement. Blood analyses did not have abnormalities, and HIV was negative.

**Figure 1:**
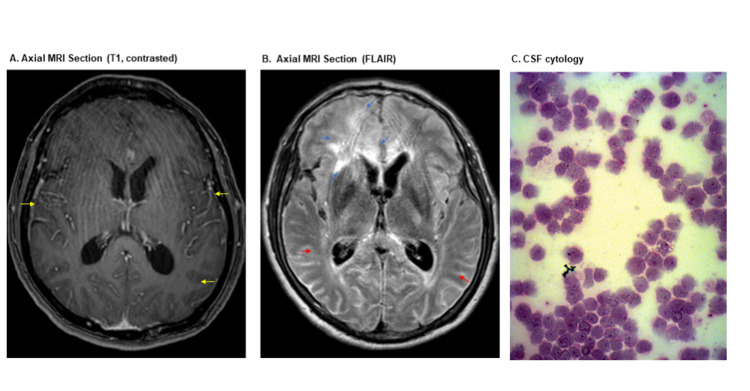
MRI images and CSF cytology Panel A: Yellow arrows indicating leptomeningeal enhancement Panel B: Blue arrows indicating right frontal lobe hyperintensity without contrast enhancement or decreased diffusion (not shown). Red arrows demonstrate hyperintensity on subarachnoid space. Panel C: Cytospin. Wright stain: 40X

A new brain MRI showed leptomeningeal enhancement (Figure 1, Panel A). Fluid-attenuated inversion recovery (FLAIR) had a hyperintensity of subarachnoid space predominantly in the convexity of both cerebral hemispheres and an ill-defined hyperintensity of the right frontal basal white matter; this lesion did not restrict diffusion and no enhancement of the parenchyma ( Figure 1, Panel B).

The new CSF flow cytometry (Table 2, Columns B and C) found a clonal B-cell population with CD20 expression, with atypical large cells in the cytospin preparations (Figure 1, Panel C) consistent with primary leptomeningeal B-cell lymphoma. A leptomeningeal biopsy was not performed, considering the flow cytometry results.

The patient received dexamethasone and HD-MTX (3.5 g/m^2^) every two weeks with rituximab (375 mg/m^2^) on days -5 and 0. After two cycles, the clinical condition improves with persistent visual loss at the time of discharge. Radiation therapy was not performed because of the rapid clinical improvement. He continues treatment in the ambulatory clinic and, the last CSF was normal without clonal cells detected in flow cytometry (Table 2, Column D).

## Discussion

The diagnosis of PLML requires (a) no prior diagnosis or subsequent discovery of systemic lymphoma; (b) no prior organ transplantation, immunosuppressive therapy, systemic or intrathecal antineoplastic drug therapy, or acquired immune deficiency syndrome; (c) no parenchymal or subependymal CNS lesion on head images (MRI or CT) with contrast enhancement; (d) negative staging evaluation for systemic lymphoma [1], and demonstration of malignant lymphocytes on CSF cytology or flow cytometry [3].

As in our patient, CSF analyses found hypoglycorrhachia (54%), elevated protein (92%), and lymphocytic pleocytosis (92%) [2]. High adenosine deaminase activity (ADA) sets differential diagnoses with other conditions like infections (tuberculosis, cryptococcosis, and syphilis), sarcoidosis, and carcinomatosis [4]. Adenosine deaminase is an enzyme involved in the catabolism of purine bases, forming inosine in the process; its main physiologic activity is related to lymphocytic proliferation and differentiation [5]. Lack of evidence of ADA in CSF cut-off and the overlap in performance of the assay affect its interpretation, missing differential diagnosis like PLML [4].

Whole-body enhanced computed tomography (CT) and fluorodeoxyglucose positron emission tomography (FDG-PET) are necessary to rule out systemic lymphoma [3].

In the case series by Taylor [2], the median age at the diagnosis was 51 years and was more frequent in men, like our patient. The time to diagnosis was two months. The clinical presentations include cranial neuropathies (50%), predominantly VI and VII cranial nerves palsy, headache (44%), ataxia (25%), and encephalopathy(25%) [2]. The presentation with blindness as seen in our patient was less frequent, with one case reported in the series of Lachance [1]. The proposal pathophysiological mechanisms of blindness include chronic papilledema, direct lymphoma infiltration, and meningeal tumor-cuffing.

The MRI findings in our patient were typical. In one reported series, PLML patients have abnormal MRI findings in 81%, with thickening and leptomeningeal enhancement, especially of the visible cranial nerve [2]. Additionally, we observed subarachnoid FLAIR hyperintensity and a prolonged signal on T2 in the frontal lobe parenchyma without contrast enhancement or decreased diffusion. These changes represent reactive edema secondary to venous congestion caused by the overlying leptomeningeal tumor that compromises cortical veins and perivascular spaces [6]. These neuroimaging findings explain altered consciousness and blindness by meningeal tumor-cuffing.

The diagnosis of PLML requires a CSF cytology suggestive of lymphoma with sharp nuclear notches, irregular cytoplasm, and increased cell size (2.5 times the upper limit of normal) [3]. The sensitivity of CSF cytology varies widely (2%-32%). This sensitivity increases when combined with flow cytometry [3]. Taylor et al. found abnormal CSF cytology in 67% and pathologic flow cytometry in 80% of PLML patients [2].

Early treatment after diagnosis is essential. HD-MTX alone or combined with rituximab, temozolomide, and other agents (cytarabine and thiotepa) are used for induction [7]. Autologous stem cell transplantation can be considered for consolidation in young and fit patients [8].

Whole-brain and spinal radiotherapy are associated with the highest risk of leukoencephalopathy and cognitive impairment [8-9], which is an option in refractory or relapsed settings.

The prognosis has improved with the introduction of HD-MTX treatment, with a median overall survival of 24 months in the most recent series [2].

## Conclusions

In conclusion, PLML can present with a myriad of neurological symptoms that include blindness and encephalopathy. MRI findings and CSF analysis with cytology and flow cytometry are necessary for a diagnosis. PLML mimics other conditions, and CSF tests like ADA activity can lead to suspicion for infectious disease, with delay in the appropriate treatment. A complete workout to exclude systemic lymphoma, including a bone marrow trephine biopsy, is necessary. PET-CT is the preferred image modality because of the higher sensibility to detect systemic lymphoma over CT scan. In the absence of clinical trials that include only patients with this disease, treatment is similar to other forms of primary central nervous system lymphoma, with high-dose methotrexate alone or in combination with rituximab and other agents. The role of high-dose chemotherapy with autologous stem cell transplantation is unclear but is an option in young and fit patients.
